# Wild boar: an increasing concern for Aujeszky's disease control in pigs?

**DOI:** 10.1186/1746-6148-8-7

**Published:** 2012-01-17

**Authors:** Mariana Boadella, Christian Gortázar, Joaquín Vicente, Francisco Ruiz-Fons

**Affiliations:** 1 IREC (CSIC-UCLM-JCCM), Ronda de Toledo s/n, Ciudad Real, Spain

**Keywords:** Disease control, Monitoring, Pseudorabies, Seroprevalence, *Sus scrofa*, Wildlife

## Abstract

**Background:**

The goal of this study was describing the temporal evolution of Aujeszky's disease virus (ADV) contact prevalence among Eurasian wild boar (*Sus scrofa*) populations under different management regimes and contact likelihoods with domestic pigs. Given the recent increase in wild boar abundance throughout Europe, we hypothesized that wild boar contact with ADV would remain stable in time even after significant reduction of ADV prevalence in domestic pigs.

**Results:**

Sera from 1659 wild boar were collected from 2000 to 2010 within 6 areas of the Iberian Peninsula and tested for the presence of antibodies against ADV by ELISA. According to sampling date, wild boar were grouped into three time periods. ADV prevalence was compared through period both globally and by geographic area. Overall seroprevalence for the ten-year study period was 49.6 ± 2.4%. The highest seroprevalence was recorded in areas with intense wild boar management. The annual proportion of positive wild boar sampling sites remained stable through the study period, while the percentage of domestic pig AD positive counties decreased from 70% in 2003 to 1.7% in 2010.

**Conclusions:**

Results presented herein confirmed our hypothesis that ADV would remain almost stable in wild boar populations. This evidences the increasing risk wild boar pose in the final stages of ADV eradication in pigs and for wildlife conservation.

## Background

Aujeszky's disease (AD), also known as pseudorabies, is one of the most economically important infectious diseases of swine for which suids are the natural hosts [1]. The disease is caused by Suid herpesvirus type I, a neuroinvasive virus with a wide host range that excludes only higher primates. Mammals other than suids are considered dead-end hosts since infection is normally fatal before virus excretion occurs. AD has a high economic impact in pig husbandry, both through direct effects of the disease on the animals and through movement and trade restrictions of pigs and their products. The direct impact of AD in wild boar population dynamics is considered to be low, but AD outbreaks with associated wild boar mortality have been reported and restrictions to wild boar movements may also have an impact on wild boar production for hunting [2,3].

Implications in conservation are considerable since fatal cases have repeatedly been described in endangered carnivores after consumption of ADV contaminated meat [4,5]. In the Iberian Peninsula, the Iberian wolf (*Canis lupus signatus*) uses Eurasian wild boar (*Sus scrofa*, the ancestor of the domestic pig) as an important part of the diet [6]. From the literature reviewed, to date ADV infection has not been reported in wolves even though fatal cases do occur in hunting dogs [7]. Moreover, other endangered carnivores such as the brown bear (*Ursus arctos*) and the Iberian lynx (*Lynx pardinus*) do occasionally consume wild boar among their prey or carrion species [8,9], and thus may also be at risk of ADV infection (e.g. fatal ADV reports in brown bears [5,10]).

Wildlife can act as reservoirs for pathogens shared with their related domestic species, being able to transmit and maintain them even without the presence of the domestic reservoir [11]. The wild boar-domestic pig interface represents one of the clearest examples of this scenario, as both species have a mutual transmission risk for their infectious and parasitic diseases [2,12]. As disease eradication programs are implemented in the domestic species, wildlife reservoirs should be considered for the program success since they come to be increasingly important [13].

In many parts of the world, efforts are being carried out to control ADV in domestic pigs. In Europe, most countries (including Spain) have implemented strict national eradication programs based on initial large scale vaccination of pigs with attenuated glycoprotein E (gE)-deleted vaccines. In countries that have reached the AD-free status, vaccination against ADV is forbidden [14]. But despite the efforts and subsequent success on AD eradication in domestic pigs, the disease is being continuously reported in wild boar populations. For instance, Germany achieved the AD-free status in 2003 despite the increasing seroprevalences (from 0.4% in 1985 to 16.5% in 2008) and widespread AD distribution in wild boar [14,15]. In France, occasional outbreaks have been described in outdoor pig farms, where contact with wild boar was deemed as the origin [16,17]. ADV contact prevalence in wild boar has also been recorded in several other European countries, such as Spain (0.8-44% [18,19]), France (3.5% [20]), Italy (30-51% [21,22]), Switzerland (2.8% [23]), Croatia (55% [24]), Slovenia (31% [25]), Poland (11% [26]) and Russia (32% [27]); suggesting that ADV may be endemic in most of these wild boar populations. In contrast, countries with limited wild boar populations such as Netherlands, or Sweden with recently expanding wild boar populations, do not record ADV in wild boar [28,29].

In Spain, the national AD eradication scheme started in 1995 (Royal Decree [RD] 245/1995) [30]. The main control measures were compulsory vaccination with gE negative vaccines, movement restriction and serological testing. The AD eradication program was reinforced in 2003 (RD 427/2003) and subsequently in recent years by applying tighter animal movement restrictions and more intensive serological testing and vaccination [31]. The AD eradication program has led to a considerable reduction of ADV prevalences in domestic pigs, although eradication in the whole territory has not yet been achieved [30].

The wild boar is the most widespread and generally also the most abundant wild ungulate in large portions of the Iberian Peninsula. Wild boar populations are continuously expanding numerically and geographically [32,33]. Furthermore, in some areas of the south-central Iberian Peninsula, wild boar are part of a growing hunting industry where management practices, such as high-wire fencing, artificial feeding and restocking are on the rise [32]. At the same time, sanitary measures for wildlife are not being implemented to match this development. As a result, high wild boar densities have already been shown to be a risk factor with negative consequences for the control of AD and other infectious diseases [34-37].

Although for ADV it has been shown that the prevalence in wild boar populations was not a significant risk factor for the level of AD prevalence in the coexisting pig farms [37], there are studies that suggest the opposite [38]. Moreover, the experimental infection of domestic pigs with ADV strains of wild boar origin [39] and the excretion of virus to the environment by wild boar [40,41], suggest the possibility of ADV transmission between both suids.

The goal of this study was describing the temporal evolution of ADV contact prevalence among wild boar populations under different management regimes and varying contact with pigs in Spain. Based on the European literature, we hypothesized that wild boar contact with ADV would remain stable in time even after significant reduction of ADV prevalence in domestic pigs.

## Results

**Figure 1:**
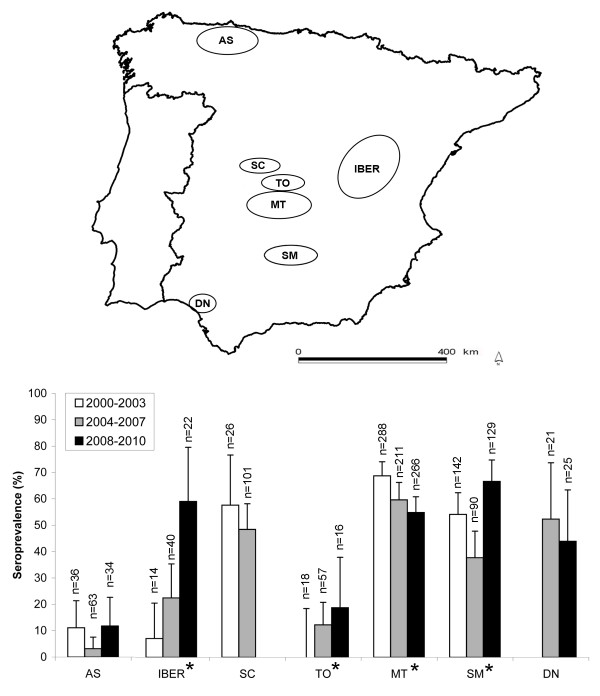
**Sampled areas and Aujeszky's disease virus (ADV) seroprevalences**. Map of the Iberian Peninsula showing the six sampled areas for the study (upper panel). Seroprevalences (and associated 95% standard errors) for each area during the three considered seasons (2000-2003, 2004-2007, 2008-2010) are shown in the lower panel. Within each area, significant differences in overall season seroprevalence are marked with an asterisk.

The overall seroprevalence for the ten-year study period was 49.6 ± 2.4% (S.E. at 95% CI), (Rogan-Gladen correction [RGC]: 50.7 ± 2.4). Antibody prevalences were high in all areas except for AS (7.5 ± 4.4% [RGC: 4.9 ± 3.7%]) and TO (11 ± 6.4% [RGC: 8.7 ± 5.8%]). Figure 1 shows the observed prevalences by area in the three sampling periods. The highest mean seroprevalences were recorded in areas where intense wild boar management was present: MT (61.4 ± 3.4% [RGC: 63.5 ± 3.4%]) and SM (54.6 ± 5.1% [RGC: 56.1 ± 5.1%]).

In three areas the observed increase in seroprevalence was statistically significant (IBER, TO and SM), while in MT the seroprevalence decreased (Chi-square, p < 0.005 in all cases). In TO area, ADV contact appeared for the first time in the period 2004-2007 (12.3 ± 8.5% [RGC: 10.1 ± 7.8%]) and increased in the following period (Figure 1).

**Figure 2:**
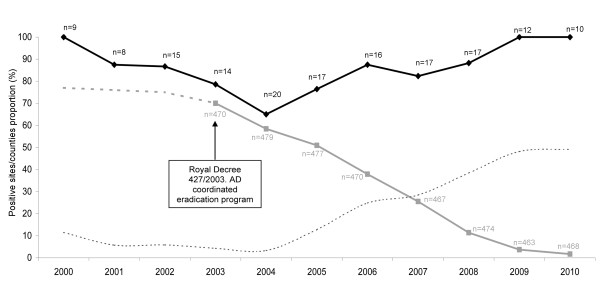
**Temporal trends on Aujeszky's disease virus (ADV) seroprevalences in wild boar and pig**. Time trend (2000-2010) of the annual proportion of sampling sites with seropositive wild boar (black diamonds), and of the proportion of counties in Spain with ADV in domestic pig (grey squares; based on data from the Spanish Ministry of the Environment and Rural and Marine Affairs, MARM). Numbers on the black line indicate the number of wild boar sampling sites per year. Numbers in grey indicate the number of reported counties per year. The discontinuous grey line is an estimated prevalence of positive counties before 2003 as data were not available before this date. The dotted line represents the hypothetical relative risk of ADV spill-back from wild boar to domestic pig, based on the difference between the pig and wild boar ADV proportions.

The annual proportion of individual sampling sites with at least one seropositive wild boar remained stable during the ten-year period, while the percentage of domestic pig AD positive counties decreased from 70% in 2003 to 1.7% in 2010 (Figure 2).

**Figure 3:**
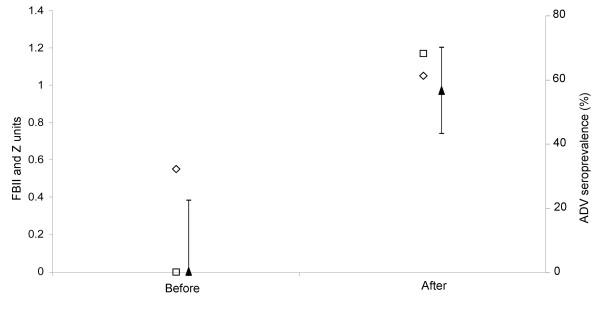
**Aujeszky's disease virus (ADV) seroprevalence and wild boar relative abundance and spatial aggregation changes in a private hunting estate**. Wild boar relative abundance (FBII; diamonds), aggregation index (Z; squares), and ADV seroprevalence (black triangles, 95% CI) in an estate where wild boar management drastically changed during the study period.

In one specific study site in northern Spain, outside the range of the main high prevalence areas, ADV seropositivity was first detected in 2008 in 27 out of 48 sampled wild boar (56.3 ± 14% [RGC: 57.9 ± 14%]). The estimated level of confidence for the negative results in the preceding period 2003-2007 (none out of 12) was 95% for an expected prevalence of 20%. Wild boar censusing confirmed a marked increase in abundance and spatial aggregation between both time periods (Figure 3).

## Discussion

Results presented here confirmed our hypothesis that ADV would remain almost stable in wild boar populations. This occurred in those areas where wild boar production as a hunting resource is practiced, and ADV seroprevalences are high. Results also showed increasing seroprevalence rates for some of the studied areas in spite of the decreasing trend reported in pigs. Time trends in wild boar contact with ADV were independent of the area's likelihood of contact with pigs, adding evidence to the hypothesis of that AD maintenance in wild boar is independent of the pig situation [14,37,41].

Sample sizes per individual site were small. This motivated studying representative areas for wild boar distribution and management characteristics in Spain. The limited sample size also means that results, particularly regarding time trends by area, need to be taken with caution. However, total wild boar ADV seroprevalence clearly remained stable after ten years, confirming that AD remains endemic at high prevalences in the south-central Spanish wild boar populations [18,37]. This finding is in compliance with other studies that record stable or even increasing trends of ADV contact in different wild boar and feral pig populations [14,15,20,42]. In our area, wild boar density and spatial aggregation within fenced hunting areas have been previously identified as risk factors for wild boar ADV contact prevalence [35,37]. These factors have not changed during the studied period. Thus, in the absence of any control measure and considering the ability of ADV to remain latent in infected suids [43], ADV prevalences were not expected to decline. Prevalences recorded in areas with intense management are among the highest of the literature worldwide [1]. Thus, the observed time trends in these prevalences (decrease in MT and increase in SM) may represent cyclic fluctuations around a "steady state" that ADV seroprevalence may have reached under these particular conditions. Even though wild boar population characteristics are different, a similar dynamic situation has also been proposed to be occurring in wild boar ADV high-prevalence areas of Germany [1]. This asymptote seems not to have been reached in other Spanish wild boar populations. Furthermore, intense hunting management practices are becoming popular in certain areas outside south-central Spain. This might suggest that higher prevalences will be reached as the wild boar population increases and the management becomes more intense.

The specific case illustrated in Figure 3 is an example of the effect of intense wild boar management for hunting on the temporal trend of ADV seroprevalence. Fencing and feeding led to a significant increase of wild boar abundance and aggregation [35], and to the detection of high contact prevalences with ADV (56%). It is unlikely that a high ADV prevalence could have gone undetected in the preceding period. Therefore, based on the current and previous observations [18], we suggest that the emergence of ADV seroprevalence could be boosted by intense hunting management practices, including a possible translocation of wild boar from positive sites. As suggested for tuberculosis, efforts should be done to control the proliferation of such intense game management without sanitary control in disease-free areas, since they can become risk hotspots with negative implications for animal health and for conservation [44].

In contrast and despite of the situation in the studied wild boar populations, ADV seroprevalence in Spanish domestic pigs experienced a significant reduction, thus showing that the eradication efforts were successful. A comprehensive study of European ADV isolates of wild boar origin, including Spanish ones, demonstrated that all except one belonged to genotype I [45]. Based on the observation that mainly type II strains were found in domestic pigs in Central Europe, it has been suggested that infections of wild boar by domestic pigs did not occur recently [1]. Hence, spill over between pigs and wild boar is apparently not a frequent event. However, the pig vaccination campaigns probably had a main role in this decrease of ADV, but we open the question of which will be the situation if Spain reaches the ADV-free status and pig vaccination is no longer permitted? Outdoor pig production is an environmentally friendly and sustainable productive system that additionally improves animal welfare and product quality, aspects that are increasingly demanded by the European society. These added values of outdoor production carry nonetheless a sanitary risk because of the increased probability of interactions with wild boar and other wildlife of uncontrolled sanitary status. There are several examples in the literature about the link between open-air or back-yard pig production and the risk of disease transmission at the pig-wild boar interface (Classical Swine Fever in Germany [46]; African swine fever in Sardinia [47] and the Caucasus [48], and ADV in France [16]). Thus, when pig biosafety measures are insufficient to avoid contact with wild boar, the wild boar could become a risk for ADV re-introduction [23]. If wild boar are seen as a source of the disease, a potential conflict on biosafety can arise between the pig industry and hunting land owners [37,49]. Because of the huge difficulties in controlling ADV in free-roaming wild boar, the main recommendation to maintain ADV-free open-air produced domestic pigs would be not to stop vaccination. Nonetheless, in countries without vaccination such as Switzerland, it has been advised to include outdoor pigs in areas at risk in routine wild boar ADV surveillance programs, since transmission between infected wild boar and outdoor pigs might occur in the future [23]. In parallel, it is important to drive efforts towards improved pig biosafety [50], along with continuous monitoring of the wild boar AD epidemiological situation (e.g. the recent establishment of the Spanish National Wildlife Disease Surveillance Scheme [51]). Eventually, research on means to control ADV in wild boar could be pertinent [52].

In the Iberian Peninsula, the presence of ADV in wild boar also exposes endangered wild carnivores to the risk of contracting lethal infection [53]. ADV contact has been detected in wild boar in protected areas where they coexist with endangered carnivores (bear and wolf in AS, wolf in IBER, wolf and lynx in SM, lynx in DN). This adds interest to ADV regarding conservation. Unfortunately, conservation programs often underestimate the role that wildlife diseases can play in their success [54].

## Conclusions

With the presented scenario, where wildlife populations represent a potential sanitary risk for livestock, trans-disciplinary wildlife disease research may provide an opportunity for stakeholders to reconsider the current approach of disease eradication in livestock towards a less severe but more sustainable concept of disease control, at least for open-air systems.

## Methods

### Wild boar sampling

A total of 1659 serum samples collected between 2000 and 2010 from beating or Monteria hunter-harvested wild boar, were selected for this retrospective study. Monteria hunting of wild boar is random and thus, is accepted as a random survey method for wild boar [55]. The selected sample was stratified by sex and age classes. Sex was known for 1503 animals and included 687 males and 816 females. Age classes of biological meaning included juveniles (n = 316), yearlings (n = 464), and adults (n = 733), as described in previous studies [56] and in Sáenz de Buruaga et al. (1991) [57]. Sera selected for this study had gone through less than five freeze-thaw cycles and severely haemolysed samples were excluded [58].

Samples came from 37 sites (range 5 to 111 samples per site) and were grouped into six geographic areas of biological meaning (Table 1; Figure 1) plus an isolated fenced estate (not shown in Figure 1). The selected areas are representative of a gradient of situations from an intense hunting management (involving fencing, artificial feeding and watering) to a lesser or inexistent hunting management. More precise descriptions of these areas have been given by [56]. One area (SM) is part of the geographical range of Iberian pig production, a traditional breed that is reared by open air farming or as backyard production (Table 1).

**Table 1:** Sample size and wild boar population characteristics of the 6 study areas.

Area	Number wild boar sampled	Wild boar density	Wild boar management	Likelihood of contact with open air raised domestic pigs
Asturias (AS)	133	Medium	Low or inexistent	Low
Sistema Central (SC)	127	Medium	Low or inexistent	Low
Sistema Ibérico (IBER)	76	Low	Low or inexistent	Low
Toledo (TO)	91	Low	Low or inexistent	Low
Montes de Toledo (MT)	765	High	Frequently intense	Low [37]
Sierra Morena (SM)	361	High	Frequently intense	High [62]
Doñana (DN)	46	Medium-high	Inexistent	Low

Number of sampled wild boar, categorized wild boar density (low, medium, high), wild boar management (inexistent to intense) and likelihood of contact with open air raised domestic pigs (low, medium, high) generally present in each of the six areas of the study, mainly based on observational data from the authors (unpublished results).

In order to analyze prevalence changes in time, samples were grouped by area into three periods: years 2000-2003, 2004-2007 and 2008-2010. We also used the annual proportion of positive sampling sites to compare with pig data on positive counties (Figure 2).

In one private hunting estate outside the described areas, we recorded wild boar relative abundance (FBII) and aggregation index (Z) in 2002 and 2010, as described in previous studies [35,56]. ADV seroprevalence was calculated for wild boar sampled in 2003-2005 (n = 12) and in 2008-2010 (n = 48). Wild boar management started to change late in 2005 through improved fencing and supplementary feeding.

### ELISA test

A commercially available blocking ELISA was used for screening of antibodies to ADV in accordance with the manufacturers' instructions (IDEXX HerdCheck Anti-ADV gpI, IDEXX, Inc., USA). This ELISA technique has been broadly used in wild boar [14,18,59] and for domestic pigs it has a sensitivity of 95-98% and a specificity of 97-99% according to the manufacturer.

### Data on pig status

ADV seroprevalence data of the control and eradication campaign in Spain at county level from 2003 to 2010 were available from the Spanish Ministry of the Environment and Rural and Marine Affairs [30]. With the data provided, we calculated the annual proportion of positive counties.

### Statistics

Standard errors at 95% confidence intervals were calculated for apparent prevalences. Mean prevalence estimates were adjusted for test sensitivity and the specificity using Rogan-Gladen corrections (RGC). RGC were calculated using the lowest values of ELISA sensitivity and specificity given by the manufacturer, 95% sensitivity-97% specificity [60]. ADV prevalences were compared through period both globally and by geographic area by means of chi-square tests. The p-value was set at 0.05. Data was analyzed using the IBM SPSS statistical package, version 19.0 (IBM Corporation, Somers, NY, USA). WinEpiscope software [61] was used to calculate the level of confidence for negative results.

## Conflict of interest statement

The authors declare that they have no competing interests.

## Authors' contributions

MB, CG, JV and FRF conceived and designed the study. MB and FRF carried out the laboratory work. MB, CG, JV and FRF participated in sampling and field work. MB and FRF analyzed the data. All authors participated in drafting the manuscript. All authors have read and approved the final manuscript.
